# Supplementary material for: A Cross-Sectional Study Investigating Canadian and Australian Adolescents’ Perceived Experiences of COVID-19: Gender Differences and Mental Health Implications
Source: Int J Environ Res Public Health. 2022 Apr 6;19(7):4407. doi: 10.3390/ijerph19074407 (PMC8998759; doi:10.3390/ijerph19074407)
Supplement: Supplementary file 1 [file ijerph-19-04407-s001.zip › ijerph-1609115-supplementary.pdf]

Supplementary Material

1. Situating the Research

It is important to situate the current research, as this cross-cultural study collected data between April and July of 2020, a few months after the WHO declared COVID-19 to be a pandemic on March 11th, 2020 [1]. As transmissions increased, governments responded by enforcing restrictions, such as quarantines, social distancing, and mandatory mask-wearing, to reduce transmissions and mortality rates [2]. These restrictions varied according to country. Therefore, the current research may provide some insight into the differences in COVID-19 experiences and psychological implications between Canadian and Australian adolescents.

At the time of testing in the province of Quebec (Canada, April to July), there were approximately 5500 (April) to 59000 (July) confirmed cases with approximately 100 (April) to 5700 (July) deaths (population ≈ 8.5 million) [3]. Conversely, in the state of Queensland (Australia), there had been a total of approximately 1100 confirmed cases and six deaths (population ≈ 5 million) [4]. In Canada, sanitary measures such as social distancing and hand sanitization were endorsed, with a 14-day obligatory quarantine if returning from outside of the country [5]. Additionally, schools were closed, indoor/outdoor gatherings were forbidden, and non-essential businesses were closed [5]. Queensland encouraged sanitary measures such as social distancing, mask-wearing, hand sanitization, and limited the number of people attending indoor/outdoor gatherings [6]. Quarantine was enforced for 14 days if returning from outside of the country or Victoria, Australia [6]. However, schools and businesses were open [6].

At the time of testing and relative to country populations, Canada reported approximately 250 (April) to 3106 (July) total confirmed cases per million and approximately 5 (April) to 237 (July) total deaths/million population [7]. Australia reported approximately 400 total confirmed cases per million and approximately four total deaths per million [7]. Towards the end of participant recruitment in June, Canada was therefore experiencing more confirmed cases and deaths per population million.

2. Double-Translation Process

The double-translation process requires two individuals: both need to be bilingual in both English and French, but one person's native language (i.e., mother tongue) is French and the other person's native language is English. The original English version of the questionnaire is translated from English to French by the francophone person. In other words, they only see the English items and they must translate them to French. Then, the anglophone receives the document but only sees the French translated items (and thus, does not see the original English items). This person's job is to translate the French items back to English. At the end of this process, both individuals compare the original English items and the items that were produced using the double-translation technique. Doing so ensures that the French items were successfully translated. If the meaning of an item is not exact/could be improved, both individuals come to a consensus on how the item could be worded otherwise in order to obtain a better translation of the original item.

Table S1. Adolescent Experiences of COVID-19 Stratified by Country and Gender.

Variable	Canada (*N*=913)		Australia (*N*=413)		Country p-value	Gender p-value
	Boys (*n* = 198)	Girls (*n* = 708)	Boys (*n* = 172)	Girls (*n* = 234)		
**Since the arrival of the coronavirus to Australia (25 January 2020), have you had symptoms (signs/manifestations) that resemble those of coronavirus (cough, fever, muscle aches, sore throat), even if they were not caused by coronavirus?**						
(0 "no symptoms" to 10 "several symptoms")	1.11 (0.83, 1.38)	1.37 (1.22, 1.52)	1.58 (1.27, 1.88)	1.85 (1.58, 2.11)	**<0.001**	**0.042**
N (Missing)	*198 (0)*	*707 (1)*	*163 (9)*	*221 (13)*		
**How have these symptoms affected your life in general?**						
(0 "it didn't affect me at all" to 10 "it affected me a lot")	0.91 (0.48, 1.33)	1.29 (1.09)	0.91 (0.58, 1.25)	1.23 (0.95, 1.51)	0.867	**0.033**
N (Missing)	*85 (113)*	*369 (339)*	*137 (35)*	*197 (37)*		
**How often have you had discussions about the coronavirus?**					**<0.001**	**<0.001**
A few times a week	81 (40.9%)	196 (27.7%)	98 (59.0%)	116 (50.9%)		
1 time a day	51 (25.8%)	179 (25.3%)	32 (19.3%)	50 (21.9%)		
2 to 5 times a day	53 (26.8%)	262 (37.0%)	26 (15.7%)	50 (21.9%)		
6 to 9 times a day	6 (3.0%)	45 (6.4%)	3 (1.8%)	10 (4.4%)		
10 times of more a day	7 (3.5%)	26 (3.7%)	7 (4.2%)	2 (0.9%)		
N (Missing)	*198 (0)*	*708 (0)*	*166 (6)*	*228 (6)*		
**Are you voluntarily following the news about the coronavirus? (Reading the newspaper, reading news on a phone, watching TV news reports)**					**<0.001**	**<0.001**
No	79 (39.9%)	168 (23.7%)	72 (44.2%)	85 (38.3%)		
Yes	119 (60.1%)	540 (76.3%)	91 (55.8%)	137 (61.7%)		
N (Missing)	*198 (0)*	*708 (0)*	*163 (9)*	*222 (12)*		

**How often (how many hours per day) do you have a look at each of the following news or media sources?**						
Traditional media (newspaper, television, radio, etc.)	0.79 (0.57, 1.00)	1.18 (1.07, 1.29)	0.53 (0.30, 0.76)	0.62 (0.42, 0.81)	**<0.001**	**0.014**
N (Missing)	*185 (13)*	*670 (38)*	*160 (12)*	*219 (15)*		
Social media news feed (Facebook, Twitter, Instagram, etc.)	1.47 (1.08, 1.86)	2.11 (1.90, 2.32)	1.72 (1.29, 2.14)	2.15 (1.79, 2.51)	0.423	**0.003**
N (Missing)	*185 (13)*	*670 (38)*	*160 (12)*	*219 (15)*		
**Websites (News.com.au, etc.)**	0.25 (0.08, 0.43)	0.44 (0.35, 0.54)	0.31 (0.13, 0.50)	0.22 (0.06, 0.38)	0.310	0.559
N (Missing)	*185 (13)*	*670 (38)*	*160 (12)*	*219 (15)*		
**Before the coronavirus arrived in Australia, how normally worried or stressed are you?**						
(1 “not at all” to 7 “a lot”)	2.88 (2.64, 3.12)	4.26 (4.13, 4.38)	2.48 (2.21, 2.74)	3.71 (3.48, 3.94)	**<0.001**	**<0.001**
N (Missing)	*198 (0)*	*708 (0)*	*158 (14)*	*218 (16)*		
**How worried or stressed are you right now?**						
(1 “not at all” to 7 “a lot”)	2.98 (2.76, 3.20)	4.01 (3.98, 4.22)	2.65 (2.40, 2.90)	3.75 (3.54, 3.96)	**0.001**	**<0.001**
N (Missing)	*198 (0)*	*708 (0)*	*158 (14)*	*218 (16)*		
**In your opinion, how much of this stress are you experiencing because of the coronavirus?**						
(1 “not at all related” to 7 “extremely related”)	3.21 (2.97, 3.45)	3.84 (3.72, 3.97)	1.82 (1.56, 2.09)	2.04 (1.82, 2.27)	**<0.001**	**<0.001**
N (Missing)	*198 (0)*	*708 (0)*	*158 (14)*	*218 (16)*		
**In the context of this pandemic (of the coronavirus being spread around the world), how worried are you about:** (1 “not at all worried” to 7 “excessively worried”)						

Your health	2.12 (1.90, 2.32)	2.74 (2.63, 2.85)	1.91 (1.68, 2.15)	2.31 (2.12, 2.51)	**0.002**	**<0.001**
N (Missing)	*198 (0)*	*707 (1)*	*159 (13)*	*220 (14)*		
Your parent's health	3.94 (3.70, 4.19)	4.24 (4.11, 4.37)	3.19 (2.92, 3.46)	3.55 (3.32, 3.77)	**<0.001**	**0.004**
N (Missing)	*198 (0)*	*707 (1)*	*159 (13)*	*220 (14)*		
The health of someone who matters to you	4.31 (4.08, 4.55)	4.92 (4.79, 5.05)	3.76 (3.50, 4.03)	4.12 (3.90, 4.35)	**<0.001**	**<0.001**
N (Missing)	*198 (0)*	*707 (1)*	*159 (13)*	*220 (14)*		
The continuation of your school year	4.01 (3.74, 4.27)	4.92 (4.78, 5.06)	2.50 (2.20, 2.79)	3.49 (3.25, 3.74)	**<0.001**	**<0.001**
N (Missing)	*198 (0)*	*707 (1)*	*159 (13)*	*220 (14)*		
Your parent's job or your job	2.61 (2.34, 2.88)	3.38 (3.24, 3.53)	2.70 (2.40, 3.01)	2.97 (2.71, 3.23)	0.222	**<0.001**
N (Missing)	*198 (0)*	*707 (1)*	*159 (13)*	*220 (14)*		
Missing something (toilet paper, medicine, fruit, bread, etc.)	1.70 (1.51, 1.89)	2.05 (1.95. 2.15)	1.71 (1.50, 1.92)	2.07 (1.89, 2.25)	0.850	**<0.001**
N (Missing)	*198 (0)*	*707 (1)*	*159 (13)*	*220 (14)*		

Note. Continuous data are presented at *M* ± 95% *CI*. Categorical data are presented as frequency (percentage). *N*/*n* = sample size. *(Missing)* = number of missing cases. Bold p-values indicate a statistically significant outcome.

Table S2. Inferential Statistics for Psychological Outcomes.

Measure	Interaction Effect (Country*Sex)	Main Effects		Simple Effects
		Country	Sex	
**SMM**	F (1, 1225) = 0.008, p = 0.930, $\eta_p^2 < 0.001$	F (1, 1225) = 19.789, $p < 0.001$, $\eta_p^2 < 0.016$<br>**Australia ↑**	F (1, 1225) = 1.909, p = 0.167, η_p^2 = 0.002	N/A
**CASI**	F (1, 1222) = 0.204, p = 0.652, $\eta_p^2 < 0.001$	F (1, 1222) = 2.519, p = 0.113, $\eta_p^2 = 0.002$	F (1, 1222) = 135.327, $p < 0.001$, η_p^2=0.100<br>**Girls ↑**	N/A
**PSS**	F (1, 1179) = 0.50.105, p = 0.246, $\eta_p^2 = 0.001$	F (1, 1179) = 8.438, p = 0.004, $\eta_p^2 = 0.007$	F (1, 1179) = 40.105, $p < 0.001$, $\eta_p^2 = 0.033$	N/A

		**Australia ↑**	**Girls ↑**	
**STATE**	F (1, 1207) = 0.454, p = 0.500, $\eta_p^2 < 0.001$	F (1, 1207) = 1.644, p = 0.200, η_p^2 =0.001	F (1, 1207) = 57.198, $p < 0.001$, η_p^2 =0.045<br>**Girls ↑**	N/A
**CTAS**	F (1, 1133) = 2.111, p = 0.147, η_p^2 = 0.002	F (1, 1133) = 9.057, p = 0.003, η_p^2 = 0.008<br>**Australia ↑**	F (1, 1133) = 79.811, $p < 0.001$, η_p^2 = 0.066<br>**Girls ↑**	N/A
**CRQ**	F (1, 1152) = 9.208, p = 0.002, η_p^2 = 0.008	F (1, 1152) = 6.616, p = 0.010, η_p^2 = 0.006<br>**Canada ↑**	F (1, 1152) = 76.420, $p < 0.001$, η_p^2 = 0.062<br>**Girls ↑**	**Boys (Canada*Australia):**<br>F (1, 1152) = 11.792, $p < 0.001$, η_p^2 = 0.010 **Canada ↑**<br>**Girls (Canada*Australia):**<br>F (1, 1152) = 0.160, p = 0.689, $\eta_p^2 < 0.001$<br>**Canada (Boys*Girls):**<br>F (1, 1152) = 21.513, $p < 0.001$, η_p^2 = 0.018 **Girls ↑**<br>**Australia (Boys*Girls):**<br>F (1, 1152) = 55.789, $p < 0.001$, η_p^2 = 0.046 **Girls ↑**
**Z-scored Depression**	F (1, 1161) = 0.111, p = 0.740, $\eta_p^2 < 0.001$	F (1, 1161) = 2.174, p = 0.141, η_p^2 = 0.002	F (1, 1161) = 68.834, $p < 0.001$, η_p^2 = 0.056<br>**Girls ↑**	N/A

Note. Arrows indicate the direction of the effect. For example, "Australia ↑" suggests that the Australian group reported a significantly greater score on that particular variable. *indicates the interaction between variables.

Table S3. Descriptive Statistics for the PSS-C Scale Items.

PSS-C Items	Canada (N = 913)	Australia (N = 413)
1. In the past week, how often did you feel rushed or hurried?	1.16 (1.08, 1.24)	1.47 (1.36, 1.57)
N (Missing)	*847 (66)*	*340 (73)*
2. In the past week, how often did you have enough time to do what you wanted?[R]	0.65 (0.57, 0.72)	1.27 (1.27, 1.37)
N (Missing)	*847 (66)*	*340 (73)*

3. In the past week, how often did you feel worried about being too busy?	0.99 (0.90, 1.07)	1.29 (1.18, 1.41)
N (Missing)	*847 (66)*	*340 (73)*
4. In the past week, how often did you feel worried about grades or school?	1.43 (1.34, 1.52)	1.56 (1.44, 1.68)
N (Missing)	*845 (68)*	*340 (73)*
5. In the past week, how often did your mum and/or dad make you feel better?[R]	1.40 (1.32, 1.48)	1.35 (1.24, 1.46)
N (Missing)	*846 (65)*	*340 (73)*
6. In the past week, how often did your mum and/or dad make you feel loved?[R]	0.67 (0.59, 0.74)	0.82 (0.72, 0.92)
N (Missing)	*845 (68)*	*340 (73)*
7. In the past week, how often did you feel scared or nervous?	1.13 (1.04, 1.21)	1.23 (1.12, 1.34)
N (Missing)	*845 (68)*	*340 (73)*
8. In the past week, how often did you feel angry?	1.33 (1.25, 1.41)	1.22 (1.12, 1.32)
N (Missing)	*845 (68)*	*340 (73)*
9. In the past week, how often did you feel happy?[R]	0.90 (0.83, 0.97)	.95 (0.85, 1.04)
N (Missing)	*845 (68)*	*340 (73)*
10. In the past week, how often did you get enough sleep?[R]	0.93 (0.85, 1.02)	1.29 (1.19, 1.40)
N (Missing)	*845 (68)*	*340 (73)*
11. In the past week, how often did you have fights with your friends?	0.29 (0.24, 0.34)	0.36 (0.30, 0.43)
N (Missing)	*845 (68)*	*340 (73)*
12. In the past week, how often did you play with your friends?[R]	1.91 (1.83, 1.99)	1.13 (1.02, 1.23)
N (Missing)	*845 (68)*	*340 (73)*
13. In the past week, how often did you feel that you had enough friends?[R]	1.04 (0.95, 1.13)	1.04 (0.92, 1.16)
N (Missing)	*842 (72)*	*340 (73)*

Note. Continuous data are presented at *M* ± 95% *CI*. *N*/*n* = sample size. *(Missing)* = number of missing cases.

References

1. World Health Organisation. Rolling Updates on Coronavirus Disease (COVID-19). 2021. Available online: https://www.who.int/emergencies/diseases/novel-coronavirus-2019/events-as-they-happen (accessed on 12.07.21).
2. World Health Organisation. Weekly Epidemiological Update on COVID-19—6 July 2021. Available online: https://www.who.int/publications/m/item/weekly-epidemiological-update-on-covid-19---6-july-2021 (accessed on 12.07.21).
3. Ministère de la Santé et des Services Sociaux. Historique du Portrait Quotidien des cas Confirmés. 2021. Available online: https://www.donneesquebec.ca/recherche/dataset/covid-19-portrait-quotidien-des-cas-confirmes/resource/d2cf4211-5400-46a3-9186-a81e6cd41de9 (accessed on 12.07.21).
4. Queensland Government. Queensland COVID-19 Statistics. 2021. Available online: https://www.qld.gov.au/health/conditions/health-alerts/coronavirus-covid-19/current-status/statistics#casesummary (accessed on 12.07.21).
5. Gouvernement du Québec. List of all Infographics Related to the Prime Minister's Announcements (COVID-19). 2021. Available online: https://www.quebec.ca/en/premier/premier/access-to-publications-related-to-the-premiers-announcements-concerning-covid-19/liste-de-toutes-les-infographies-en-lien-avec-les-annonces-du-premier-ministre-covid-19 (accessed on 12.07.21).
6. Queensland Government. Roadmap to Easing Queensland's Restrictions. 2020. Available online: https://www.torres.qld.gov.au/downloads/file/510/covid-19-restrictions-roadmap-3-july-2020 (accessed on 12.07.21).
7. Dong, E.; Du, H.; Gardner, L. An interactive web-based dashboard to track COVID-19 in real time. *Lancet Infect. Dis.* **2020**, *20*, 533–534. https://doi.org/10.1016/S1473-3099(20)30120-1.
